# Carbapenem-resistant *Morganella morganii* carrying *bla*_KPC-2_ or *bla*_NDM-1_ in the clinic: one-decade genomic epidemiology analysis

**DOI:** 10.1128/spectrum.02476-24

**Published:** 2025-03-03

**Authors:** Jiayao Yao, Yueyue Hu, Xinru Wang, Jie Sheng, Ying Zhang, Xiaofei Zhao, Jiaqing Wang, Xiufang Xu, Xi Li

**Affiliations:** 1 Laboratory Medicine Center, Department of Clinical Laboratory, Zhejiang Provincial People’s Hospital, Affiliated People’s Hospital, Hangzhou Medical College, Hangzhou, China; 2 Shaoxing Central Hospital, The Central Affiliated Hospital, Shaoxing University, Shaoxing, China; 3 School of Medical Imaging, Hangzhou Medical College 117839 , Hangzhou, China; Instituto de Higiene, Montevideo, Canelones, Uruguay

**Keywords:** carbapenem-resistant *Enterobacterales*, *Morganella morganii*, KPC-2, NDM-1, phylogenetic analysis

## Abstract

Carbapenem-resistant *Morganella morganii* (CRMM) isolates, particularly those producing Klebsiella pneumoniae carbapenemase-2 (KPC-2) or New Delhi metallo-β-lactamase-1 (NDM-1), are increasingly being recognized as causative agents of nosocomial infections. However, systematic phylogeography and genetic characterization of these isolates worldwide are still lacking. Here, through seven years of surveillance of CRMM in a tertiary hospital, we analyzed the genomic characteristics of *bla*_KPC-2_- or *bla*_NDM-1_-positive CRMM isolates. Furthermore, we conducted a global genomic epidemiological study of *Morganella* spp. harboring *bla*_KPC_ or *bla*_NDM_ using the NCBI database over the past decade. By combining the timeline of isolate collection with the structural analysis of the plasmids, we traced the evolution of the IncL/M plasmid, which acquired the *bla*_KPC-2_ gene. Our findings indicate that horizontal transfer of Tn*6296* based on IS*26* is crucial for the transmission of *bla*_KPC_ in CRMM isolates. Additionally, the Tn*125* transposon appears to have played an important role in early plasmid-mediated dissemination of *bla*_NDM_; however, it has been surpassed in recent years by other elements, including IS*26* and IS*CR*. In summary, through phylogeographic analysis of *Morganella* spp. globally, we elucidated their spatial–temporal distribution and revealed the evolutionary characteristics of KPC- or NDM-producing CRMM isolates as the predominant "epidemic" clone.

**IMPORTANCE:**

Currently, infections attributable to carbapenem-resistant *Morganella morganii* (CRMM) isolates harboring *bla*_KPC_ or *bla*_NDM_ are on the rise, highlighting the increasing severity of acquired antimicrobial resistance. However, systematic phylogeographic and genetic characterization of these isolates worldwide is still lacking. In this study, we elucidated the spatial–temporal distribution and evolutionary trajectory of *bla*_KPC_ and *bla*_NDM_ genes within their core genetic environments. We emphasize the necessity of strengthening surveillance and controlling these organisms in clinical settings to prevent the generation of so-called "superbug" isolates.

## INTRODUCTION

*Morganella* spp., belonging to the order *Enterobacterales* and family *Morganellaceae*, can be classified into six species: *Morganella morganii*, *Morganella sibonii*, *Morganella psychrotolerans*, *Morganella chanii*, *Morganella laugraudii*, and *Morganella kristinii* (1, 2). Among these, *M. morganii* is widely distributed in humans (3), animals (4), and environment (5). It has emerged as a high-risk pathogen for hospital-acquired infections worldwide, with a high incidence of urinary tract infections (UTIs) (2) and a particularly high mortality rate in patients with bloodstream infections (3). Furthermore, carbapenem-resistant *Morganella* spp. (CRMs), as part of the carbapenem-resistant *Enterobacterales*, have been categorized as a critical priority among the WHO bacterial priority pathogens, undoubtedly creating greater challenges for clinical treatment (6).

*M. morganii* is known to exhibit intrinsic resistance to various antimicrobial drugs, including ampicillin, aminopenicillins, amoxicillin-clavulanate, first- and second-generation cephalosporins, tigecycline, colistin, and nitrofurantoin (7). According to the Clinical and Laboratory Standards Institute (CLSI), *M. morganii* may have elevated minimum inhibitory concentrations (MICs) of imipenem through mechanisms other than carbapenemase production (7, 8). The therapy for infections caused by *Enterobacterales* (including *Morganella*) frequently employs β-lactams, with carbapenems being a common choice. However, the emergence of carbapenem-resistant *Enterobacterales*, particularly producing NDM-like and KPC-like enzymes, has become a significant concern (9–11). Resistance mechanisms are not limited to acquired antimicrobial resistance genes (ARGs) but also include mobile genetic elements (MGEs) (1). The frequent emergence of carbapenem-resistant *Morganella morganii* (CRMM) is primarily attributed to the spread of *bla*_NDM-1_ and *bla*_KPC-2_ (12). Since the first identification of an *M. morganii* strain carrying *bla*_NDM-1_ in an epidemiological study of clinical CRE (13), multiple strains of NDM-1-producing *M. morganii* have been isolated from various countries, including Israel (14), Brazil (15), Uruguay (12), Pakistan (16), France (17), and Nepal (18). Additionally, 10 *M. morganii* strains carrying *bla*_KPC-2_ were identified in two hospitals in China (19, 20). Notably, diverse plasmids contribute to the spread of *bla*_NDM_ and *bla*_KPC_ in *M. morganii* (21, 22) and even horizontal transfer between different species (23). However, most CRMM isolates consistently play a supporting role in large-scale epidemiological surveys of CRE (24), with only a few in-depth studies. In particular, global systematic epidemiological analysis is lacking.

In this study, we analyzed the characteristics of CRMM isolates harboring *bla*_KPC-2_ or *bla*_NDM-1_ and explored the factors that facilitate the spread of these resistance genes. In addition, a systematic epidemiological analysis of the global distribution of the CRMM isolates was performed to elucidate the underlying evolutionary links.

## RESULTS

### Characteristics of the CRMM strains

From January 2016 to December 2022, 19 CRMM strains were isolated from a tertiary hospital in Hangzhou, China. Through colloidal gold immunochromatography screening and PCR verification, six CRMM isolates containing *bla*_KPC_ (*n* = 4) or *bla*_NDM_ (*n* = 2) were obtained for further investigation. These strains were isolated from sputum and urine samples of five patients aged 55–89 years (Table S1). Antimicrobial susceptibility testing (AST) revealed that six CRMM strains were resistant to meropenem (MEM), imipenem (IPM), ertapenem (ETP), cefepime (FEP), ciprofloxacin (CIP), and ceftazidime (CAZ), but sensitive to cefiderocol (CFDC). Amikacin (AMK) resistance was observed in four strains, whereas ceftazidime-avibactam (CZA) resistance was observed in two CRMM isolates harboring *bla*_NDM-1_ (Table 1).

**TABLE 1:** Antimicrobial susceptibilities of six *M*. *morganii* strains and six transconjugants conducted by *M. morganii* and *E. coli* J53^*b*^

Isolates	Minimum inhibitory concentrations (MICs, μg/mL)									Plasmid	Gene
	MEM	IPM	ETP	AMK	FEP	CIP	CAZ	CZA^*a*^	CFDC	Type	
Clinical isolates											
MM4205	4	32	16	8	> 128	32	64	1	2	IncN (59 k)	*bla*_KPC-2_
										IncL/M (61 k)	/
MM1680	4	16	16	16	> 128	16	32	0.5	2	IncN (66 k)	*bla*_KPC-2_
										IncL/M (99 k)	*bla*_KPC-2_
MM6005	4	16	8	2	> 128	4	64	0.25	2	IncL/M (99 k)	*bla*_KPC-2_
MM4512	4	32	8	> 128	64	16	16	< 0.125	0.25	IncN (61 k)	*bla*_KPC-2_963 bp_
										IncFII-R (142 k)	*bla*_KPC-2_
MM9291	4	16	4	16	64	32	> 128	> 128	2	Unknown	*bla*_NDM-1_
MM2467	4	64	16	> 128	64	16	> 128	> 128	2	IncFII-FIB (109 k)	*bla*_NDM-1_
Transconjugants											
J53	< 0.125	0.25	< 0.125	2	0.25	< 0.125	0.25	< 0.125		/	/
J53/pMM4205-KPC-2_59k	32	4	32	4	128	< 0.125	32	0.25		IncN (59 k)	*bla*_KPC-2_
J53/pMM1680-KPC-2_66k	8	32	32	16	128	0.5	32	0.5		IncN (66 k)	*bla*_KPC-2_
J53/pMM6005-KPC-2_99k	16	4	16	2	64	< 0.125	8	0.5		IncL/M (99 k)	*bla*_KPC-2_
J53/pMM4512-KPC-2_142k	8	2	16	> 128	32	< 0.125	16	0.5		IncFII-R (142 k)	*bla*_KPC-2_
J53/pMM4512-KPC-2_142k /pMM4512-KPC-2_61k	16	8	64	> 128	128	< 0.125	64	2		IncFII-R (142k)	*bla*_KPC-2_
										IncN (61k)	*bla*_KPC-2_963 bp_
J53/pMM9291-NDM-1_257k	4	2	8	16	32	< 0.125	> 128	> 128		Unknown	*bla*_NDM-1_

^*a*^ Avibactam was added at 4 µg/mL. MEM, meropenem; IPM, imipenem; ETP, ertapenem; AMK, amikacin; FEP, cefepime; CIP, ciprofloxacin; CAZ, ceftazidime; CZA, ceftazidime-avibactam; CFDC, cefiderocol. The "/"in "Plasmid Type" indicates the absence of a plasmid; the "/" in "Gene" indicates the absence of the *bla*_KPC-2_ or *bla*_NDM-1_ gene.

^*b*^ The "/" in "Plasmid Type" indicates the absence of a plasmid; the "/" in "Gene" indicates the absence of the blaKPC-2 or blaNDM-1 gene.

Three types of plasmids (IncN, IncL/M, and IncFII-R) bearing *bla*_KPC-2_ and two types of plasmids (IncFII-FIB and an unknown plasmid) carrying *bla*_NDM-1_ were identified (Table 1). All plasmids were successfully conjugated to *E. coli* J53, except IncFII-FIB (pMM2467-NDM-1_109k). The transconjugants presented increased resistance to MEM and ETP with 4–8 times the donor’s MIC. Comparing the two transconjugants of MM4512, the transconjugant carrying double *bla*_KPC_ exhibited significantly higher levels of resistance to carbapenems than the one carrying a single plasmid with *bla*_KPC-2_.

### Stability and fitness effects of the plasmids with *bla*_KPC-2_ or *bla*_NDM-1_

**Fig 1:**
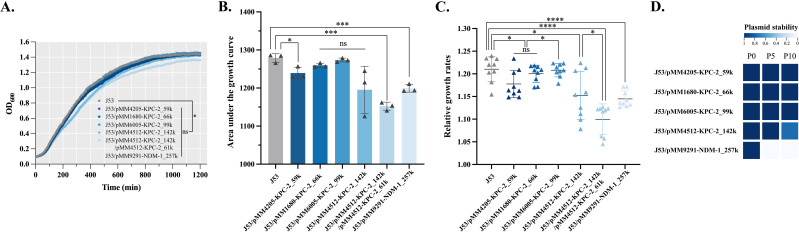
Characterization of biological features of transconjugants carrying *bla*_KPC-2_ or *bla*_NDM-1_. Based on the growth curve (**A**), the area under the curve (AUC) (**B**) and relative growth rate (**C**) were calculated and displayed as the mean standard deviation (SD). This experiment was performed in triplicate and repeated three times. ^*^P < 0.05; ^**^P < 0.01; ^***^P < 0.001; ^****^P < 0.0001 (ANOVA and two-sample *t*-test). (**D**) Plasmid stability of the transconjugants without antibiotics.

The transconjugants exhibited a significantly decreased growth rate compared with *E. coli* J53 on the growth curve (F = 2.127, *P* = 0.0476) (Fig. 1A), area under the curve (AUC) (F = 10.54, *P* = 0.0002) (Fig. 1B), and relative growth rate (F = 15.96, *P* < 0.0001) (Fig. 1C). The AUCs and relative growth rates revealed that transconjugants with IncN (pMM4512-KPC-2_61k), IncFII-R (pMM4512-KPC-2_142k), or pMM9291-NDM-1_257k (non-typable) had significantly lower growth rates (a decrease of 5.37%–9.93%, *P_t_* <0.001) than those with J53 (Fig. 1B and C). In addition, compared with a single plasmid (J53-pMM4512-KPC-2_142k) carrying *bla*_KPC-2_, two plasmids (pMM4512-KPC-2_142k and pMM4512-KPC-2_61k) carrying *bla*_KPC-2_ displayed a greater fitness cost on *E. coli* J53 (a decrease of 4.514%, *P_t_* <0.05) (Fig. 1C). Subsequently, plasmid stability revealed that the large plasmid pMM9291-NDM-1_257k was completely lost by the fifth generation in the absence of antibiotics. While most plasmids were stably inherited, one (pMM4512-KPC-2_142k) was partially lost in the progeny (Fig. 1D). Overall, these results indicate that the expression of both *bla*_KPC-2_ and *bla*_NDM-1_ genes mediated by plasmids had a negative impact on fitness.

### Genomic characteristics of CRMM strains harboring* bla*_KPC-2_ or *bla*_NDM-1_

All six CRMM isolates harbored a single chromosome (approximately 4 Mb) and carried one to three plasmids (Table S2). Various plasmids were identified in CRMM, including IncN (*n* = 3), IncL/M (*n* = 2), and IncFII-R recombinant plasmids (*n* = 1) bearing *bla*_KPC-2_ and IncFII-FIB recombinant plasmids (*n* = 1) with *bla*_NDM-1_. Notably, both the MM1680 strain (harboring IncN and IncL/M plasmids) and MM4512 strain (containing IncN and IncFII-R) possessed a single *bla*_KPC-2_ gene in their respective plasmids. Whole-genome sequencing (WGS) revealed that four *bla*_KPC-2_-bearing isolates, which contained six plasmids with *bla*_KPC-2_, possessed 22 ARGs conferring resistance to seven classes of antimicrobials (Fig. S1A through D). In contrast, two isolates carrying *bla*_NDM-1_ presented 18 ARGs, conferring resistance to six classes of antimicrobials (Fig. S1E and F).

**Fig 2:**
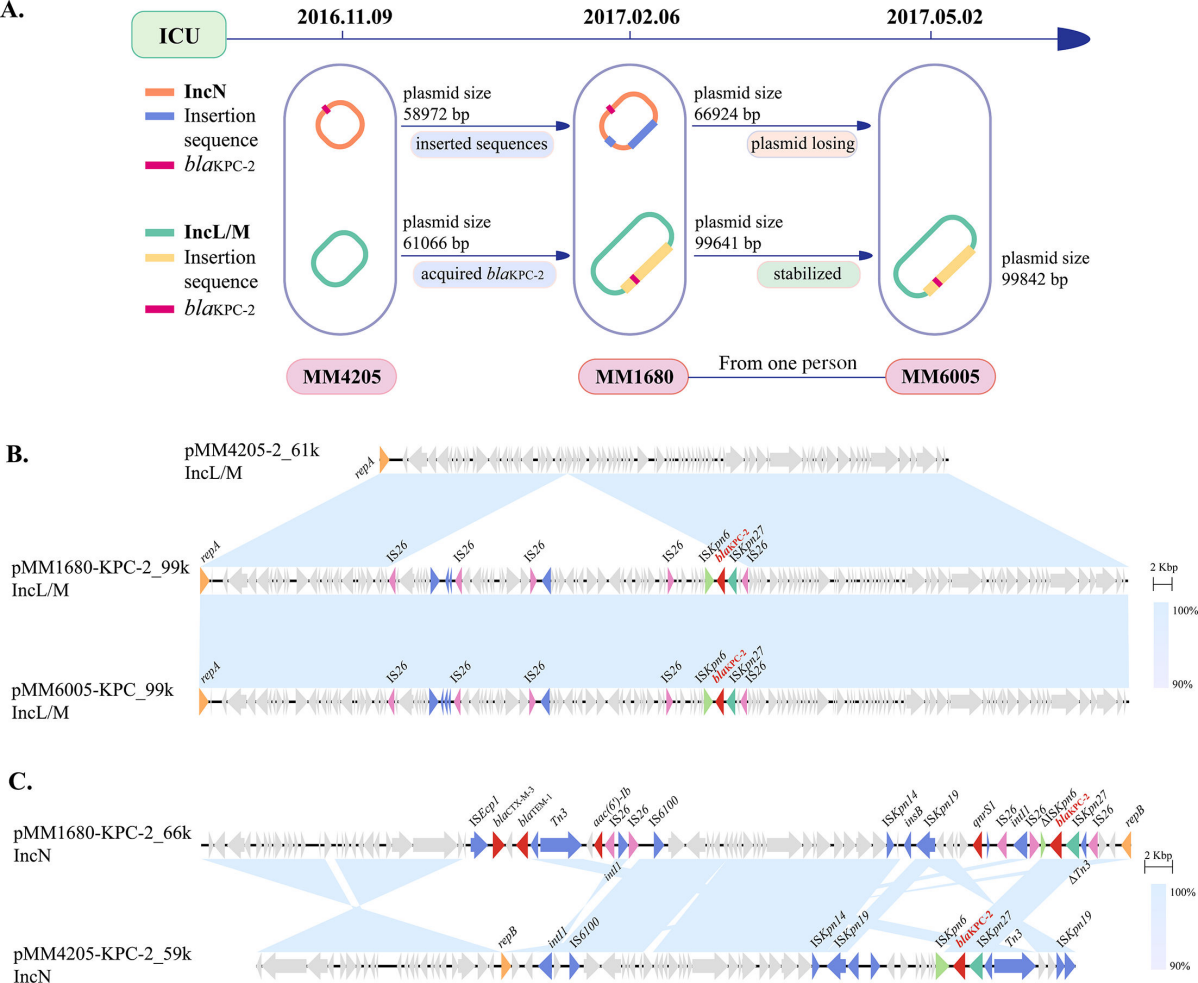
Clinical traceability of the IncL/M plasmid acquisition of *bla*_KPC-2_ facilitated by IS*26*. (**A**) Schematic diagram of IncN and IncL/M plasmid evolution in the three *M. morganii* isolates (MM4205, MM1680, and MM6005). (**B**) Comparison of three IncL/M plasmids via BLAST. This revealed the evolutionary process of *bla*_KPC-2_ acquisition in the IncL/M plasmid. Shading in light blue indicates regions of homology (nucleotide identity of ≥90%). (**C**) Evolution of IncN plasmids from pMM4205-KPC-2_59k to pMM1680-KPC-2_66k plasmids. Shading in light blue indicates regions of homology (nucleotide identity of ≥90%).

Notably, three *M. morganii* isolates were collected from two patients in the ICU, possibly revealing the evolutionary process by which the IncL/M plasmid acquired *bla*_KPC-2_ through a mobile element mediated by IS*26* (Fig. 2A through C). Single nucleotide polymorphism (SNP) identification revealed that the three isolates (MM4205, MM1680, and MM6005) were highly homologous (SNPs <25) (Fig. 6D). Second, through plasmid type and size analysis and circle diagram construction, we generated a schematic diagram of plasmid evolution combined with the timeline of the strains collected in the ICU (Fig. 2A). Finally, sequence comparison of the plasmids revealed that the sequences of pMM4205-2, pMM1680-KPC-2_99k, and pMM6005-KPC-2_99k are highly similar (Fig. 2B). However, during the evolution of IncN plasmids (from pMM4205-KPC-2_59k to pMM1680-KPC-2_66k), more fragments containing ARGs, mediated by mobile elements, were inserted (Fig. 2C).

### Events associated with the spread of *bla*_KPC_ and *bla*_NDM_ in *M. morganii*

**Fig 3:**
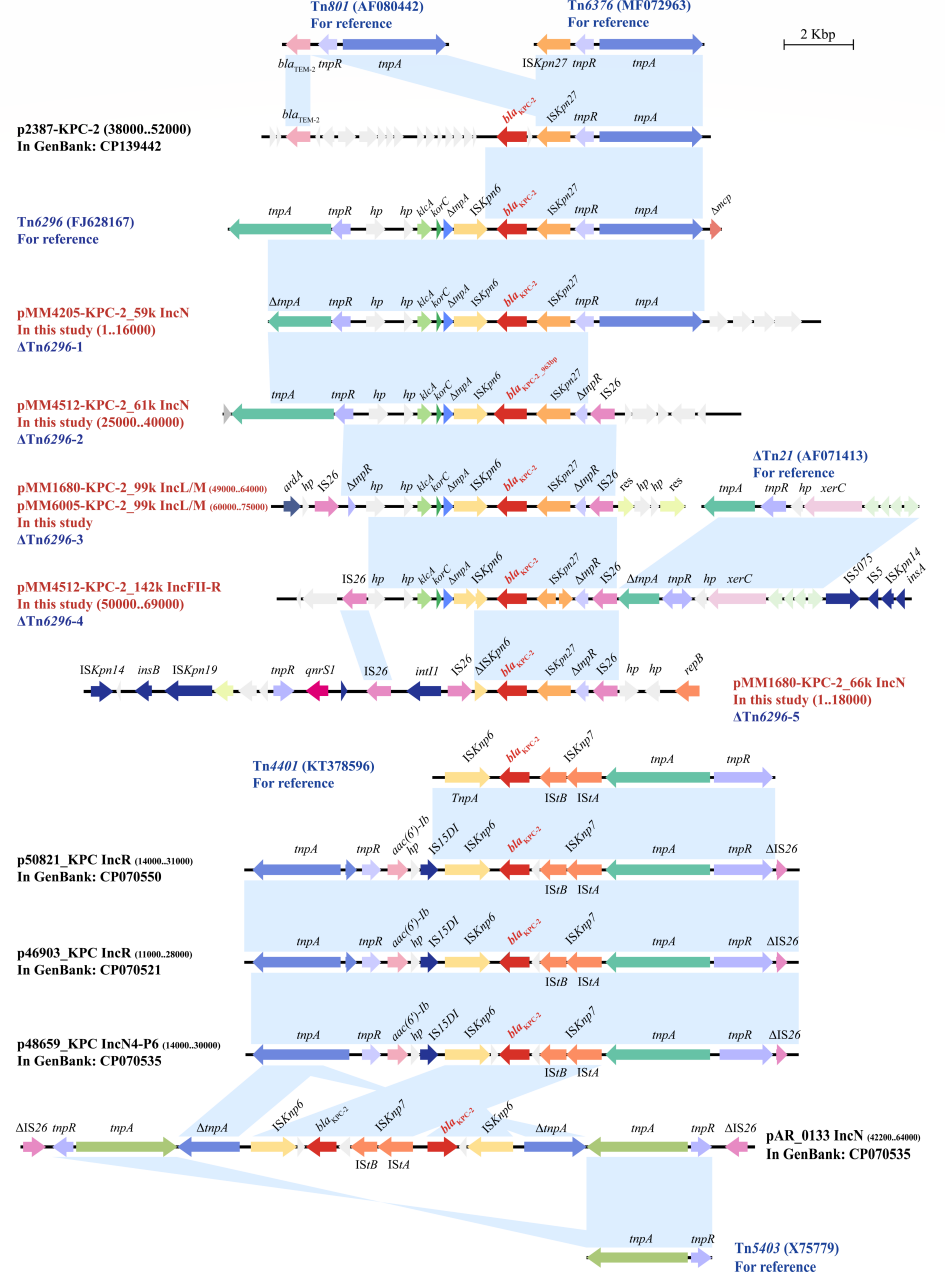
The core genetic environment of *bla*_KPC_ in *M. morganii*. The *bla*_KPC_ regions were compared with related regions. Genes are indicated by arrows. Genes, AGEs, and other features were colored according to their functional classification. Shading in light blue indicates regions of homology (nucleotide identity of ≥95%). The accession numbers of Tn*6296*, Tn*6376*, Tn*801*, ΔTn*21*, Tn*4401*, and Tn*5403* for reference are FJ628167, MF072963, AF080442, AF071413, KT378596, and X75779, respectively.

To further explore the core genetic environment of *bla*_KPC_ in *M. morganii*, we analyzed the *bla*_KPC-2_ region, which is approximately 20,000 bp in length, through sequence alignment of the six plasmids from the four strains in this study, combined with five plasmids already available in GenBank (Fig. 3). Based on the integrity of Tn*6296*, the core *bla*_KPC-2_ genetic regions in our study exhibited five groups of Tn*6296* derivatives (ΔTn*6296*-1 to ΔTn*6296*-5), primarily manifesting as insertions and deletions. ΔTn*6296*-1 (*tnpA-tnpR*-IS*Kpn27-bla*_KPC-2_-IS*Kpn6-*Δ*tnpA-korC-klcA-hp-hp-tnpR*-Δ*tnpA*) exhibited a relatively complete Tn*6296* transposon structure, whereas ΔTn*6296*-2 showed partial loss of Tn*6376* and insertion of IS*26* on the side near IS*Kpn27*. Based on ΔTn*6296*-2, ΔTn*6296*-3 lost *tnpA* on the IS*Kpn6* side, and an IS*26*-mediated sequence was inserted. ΔTn*6296*-4 was derived from Tn*6296* after the loss of truncation of Tn*6376*, along with the insertion of IS*26*, ΔTn*21*, IS*5075*, IS*5*, and IS*Kpn14*. Accompanied by the insertion of IS*26* and sequence rearrangement, ΔTn*6296*-5 showed only a few remnants of the Tn*6296* structure, specifically Δ*tnpR*-IS*Kpn27-bla*_KPC-2_-ΔIS*Kpn6*. Overall, the genetic structure IS*26-bla*_KPC-2_-IS*26* was prevalent in facilitating the spread of *bla*_KPC-2_. In addition, a Tn*4401*-like background of *bla*_KPC-2_ was discovered in p50821_KPC (CP070550), p46903_KPC (CP070521), and p48659_KPC (CP070535), and a close Tn*5403* background of *bla*_KPC-2_ was found in pAR_0133 (CP028958).

**Fig 4:**
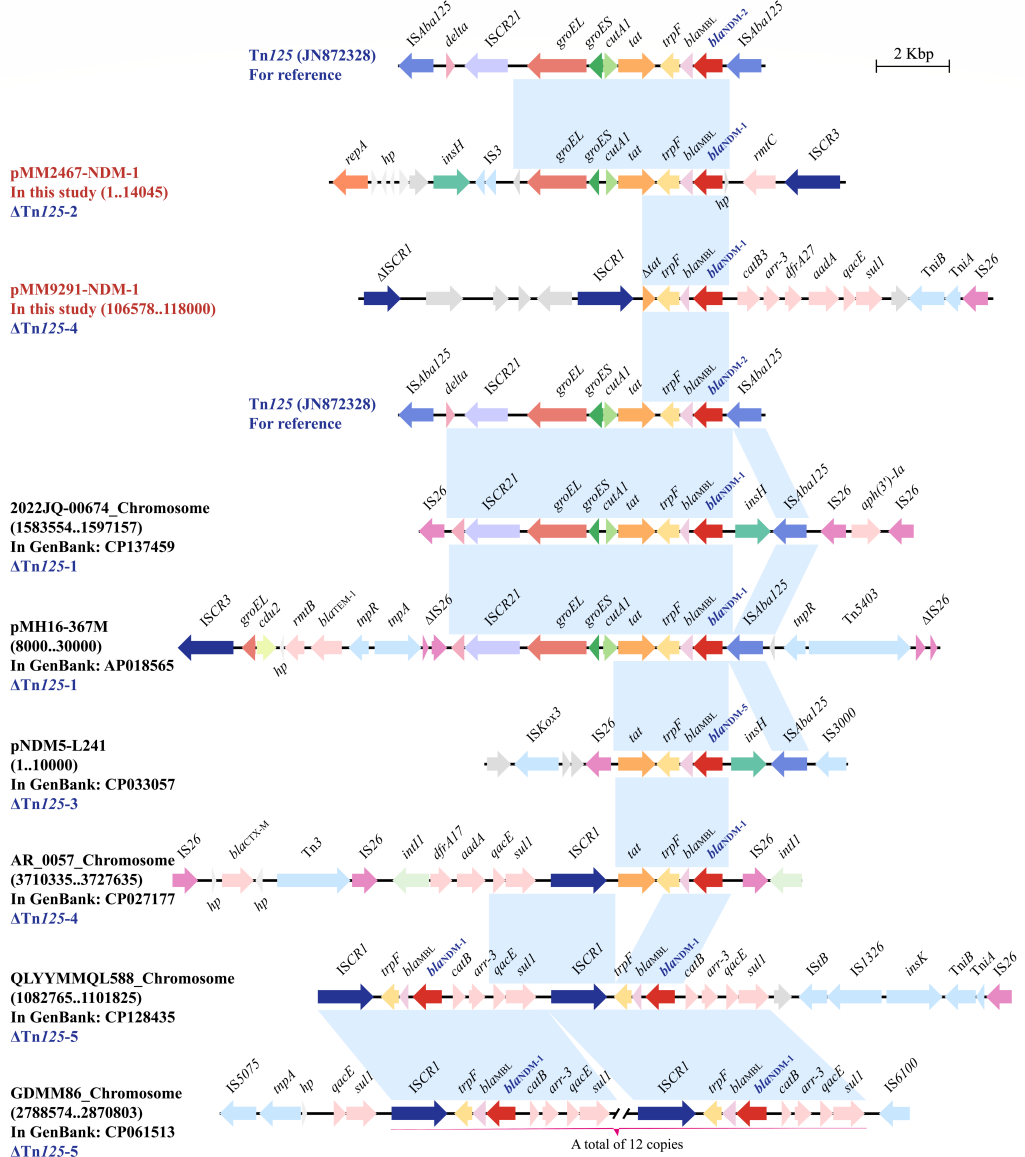
The core genetic environment for *bla*_NDM_ in *M. morganii*. The *bla*_NDM_ regions were compared with related regions. Genes are indicated by arrows. Genes, AGEs, and other features are colored according to their functional classification. Shading in light blue indicates regions of homology (nucleotide identity of ≥95%). The accession number of Tn*125* used as a reference was JN872328.

Similarly, utilizing a total of eight strains (two from this study and six from GenBank) with existing complete sequences, we conducted an analysis of the core genetic environment associated with *bla*_NDM_ (Fig. 4). The propagation of *bla*_NDM_ in a genetic context can be broadly categorized into five closely related Tn*125* derivatives (ΔTn*125*-1 to ΔTn*125*-5), with a common region (*bla*_NDM_-*bla*_MBL_-*trpF*). Overall, the upstream and downstream regions of *bla*_NDM_ displayed notable variations mediated by IS*26*, IS*CR* elements, and Tn*3* family of insertion sequences. Among them, two or 12 copies of *bla*_NDM_ located in the ΔTn*125*-5 variants (*sul1-qacE-arr-3-catB-bla*_NDM_-*bla*_MBL_-*trpF-*IS*CR1*) were integrated into the chromosomes of *M. morganii*.

### Global surveillance of KPC- or NDM-producing *Morganella* spp.

To investigate the phylogeographic relationships of *Morganella* spp., a total of 968 non-duplicate *Morganella* spp. genomes were obtained from this study (six isolates) and GenBank (962 isolates) (Table S3). Furthermore, through ARG screening, we retrieved a total of 190 (19.6%, 190/968) CRMs with *bla*_KPC_ or *bla*_NDM_, which were distributed in only three species: *M. morganii* (177 isolates), *M. sibonii* (10 isolates), and *M. chanii* (three isolates). These strains carried five variants of carbapenemases: NDM-1 (61.0%, 116/190), KPC-2 (25.2%), NDM-5 (7.8%), NDM-7 (4.7%), and KPC-3 (3.1%) (Table S4).

**Fig 5:**
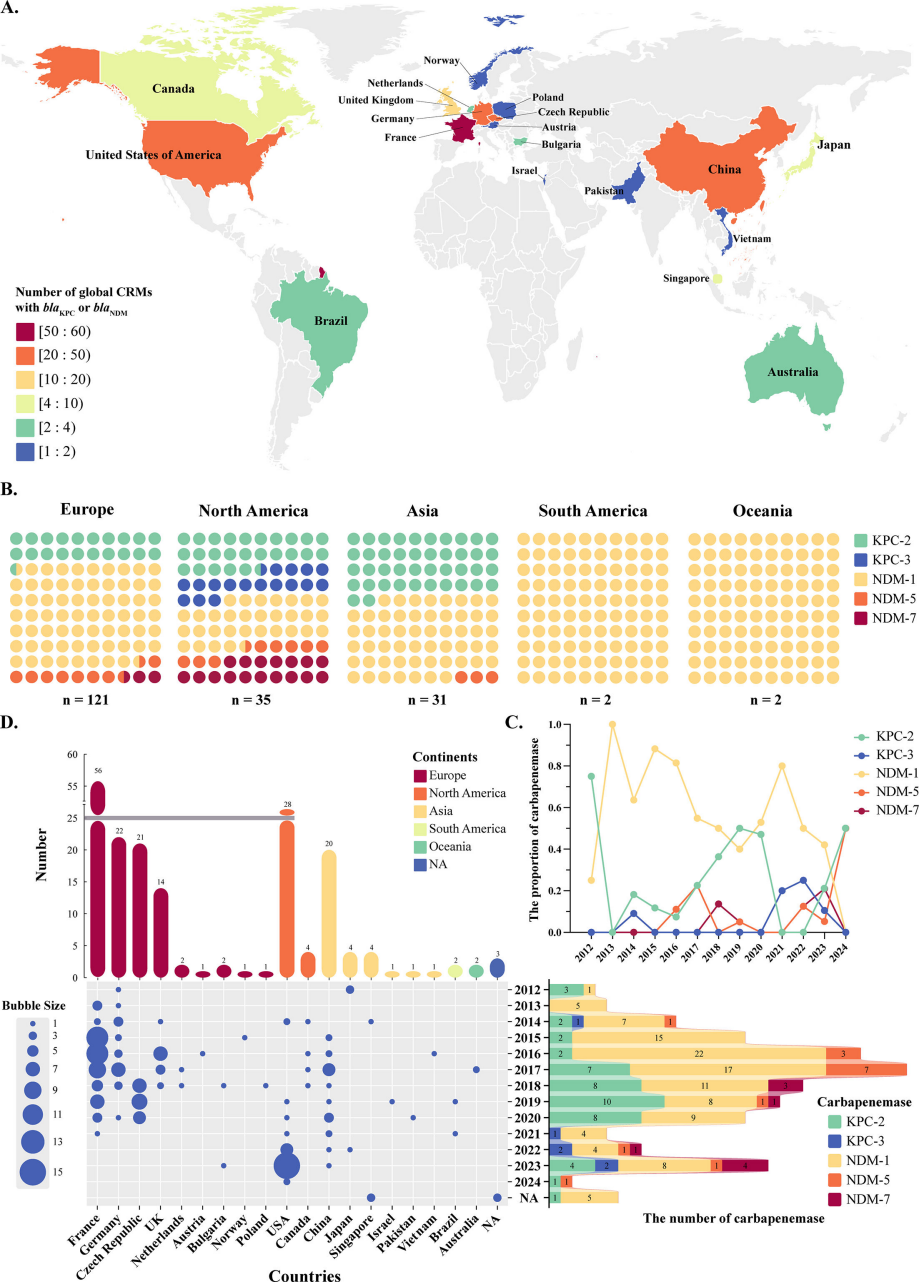
Spatial-temporal distribution of carbapenem-resistant *Morganella* spp. isolates carrying *bla*_KPC_ or *bla*_NDM_. (**A**) Global phylogeography of 181 CRMs (excluding those from three isolates unknown) in GenBank and six isolates in our study. The different colored blocks represent various quantity levels. (**B**) Proportional distribution of enzymatic phenotypes across five continents. Five enzymes (KPC-2, KPC-3, NDM-1, NDM-5, and NDM-7) are indicated by distinct colors. (**C**) Annual trend in the proportion of carbapenemases. (**D**) Timeline of *bla*_KPC_- or *bla*_NDM_-positive CRMs outbreaks in 19 countries. The bubble size was proportional to the number of strains. The bar chart illustrates the quantities and proportions of the five enzymatic phenotypes. NA: Unavailable relevant information.

Through a world map and occurrence timelines, the isolates were collected from five continents and 19 countries, primarily France (29.4%, 56/190), followed by the United States of America (14.7%), Germany (11.5%), the Czech Republic (11.0%), and China (10.5%) (Fig. 5A). Additionally, NDM-1-producing CRMs were the most widespread in the five continents, accounting for 31.43%–100%. KPC-3-producing CRMs were detected exclusively in North America, accounting for 17.14% (6/35), indicating geographic specificity in the distribution of resistance genes (Fig. 5B). An inverse relationship between the prevalence of *bla*_KPC-2_ and *bla*_NDM-1_ was observed over the past decade (Fig. 5C). In recent years, there has been an increasing trend in the emergence of *bla*_KPC-2_, warranting further investigation (Fig. 5D).

### Phylogenetic analysis of KPC- or NDM-producing *Morganella* spp.

**Fig 6:**
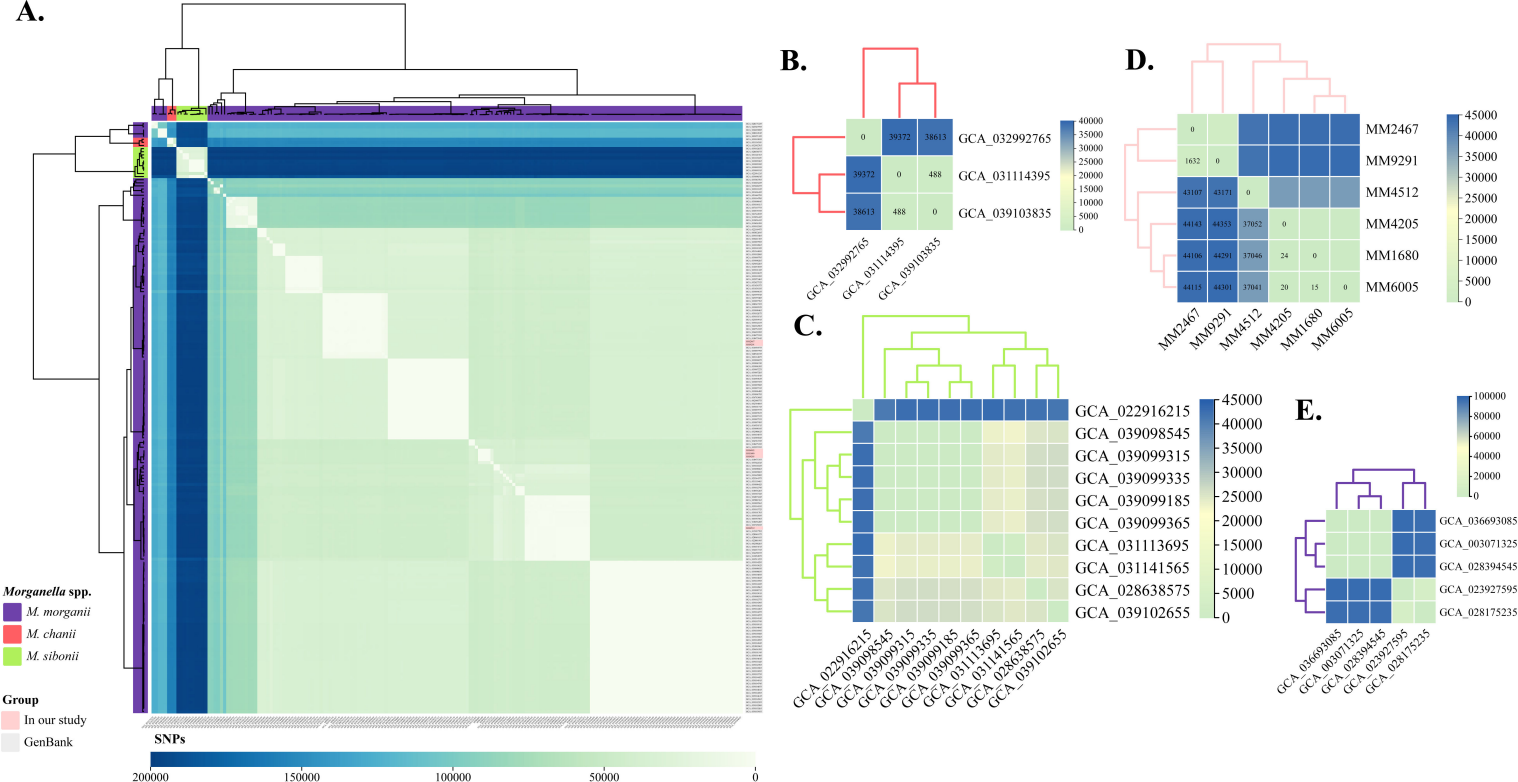
Cluster analysis of KPC- or NDM-producing *Morganella* spp. based on single nucleotide polymorphisms (SNPs). The SNP-based matrix obtained from Snippy was visualized using TBtools-II. (**A**) SNP heatmap of 190 CRMs from three species: (**B**) *M. chanii* (*n* = 3), (**C**) *M. sibonii* (*n* = 10), and *M. morganii* (*n* = 177). SNP heatmap of six *M. morganii* isolates in our study (**D**) and five *M. morganii* isolates with 95%–96% average nucleotide identity (ANI) (**E**).

To delineate the evolutionary relationships of *Morganella* spp. harboring *bla*_KPC_ or *bla*_NDM_, we analyzed the core genomes of 190 CRMs (including three species) using Snippy (Fig. 6A). In total, 195,700 SNPs were identified in these isolates. Specifically, three strains of *M. chanii* presented 488–39,372 SNPs (Fig. 6B), whereas 10 *M. sibonii* isolates presented 185–43,175 SNPs (Fig. 6C). Notably, phylogenetic divergence among the 177 strains of *M. morganii* was substantial, with SNPs ranging from 2 to 121,807. Interestingly, five *M. morganii* strains seemed to represent a distinct *M. morganii* clone, characterized by 173 to 97,402 SNPs (Fig. 6E). As shown for SNPs, we observed that several isolates from the same or different countries shared a few SNPs (<200 SNPs), suggesting possible clonal transmission.

**Fig 7:**
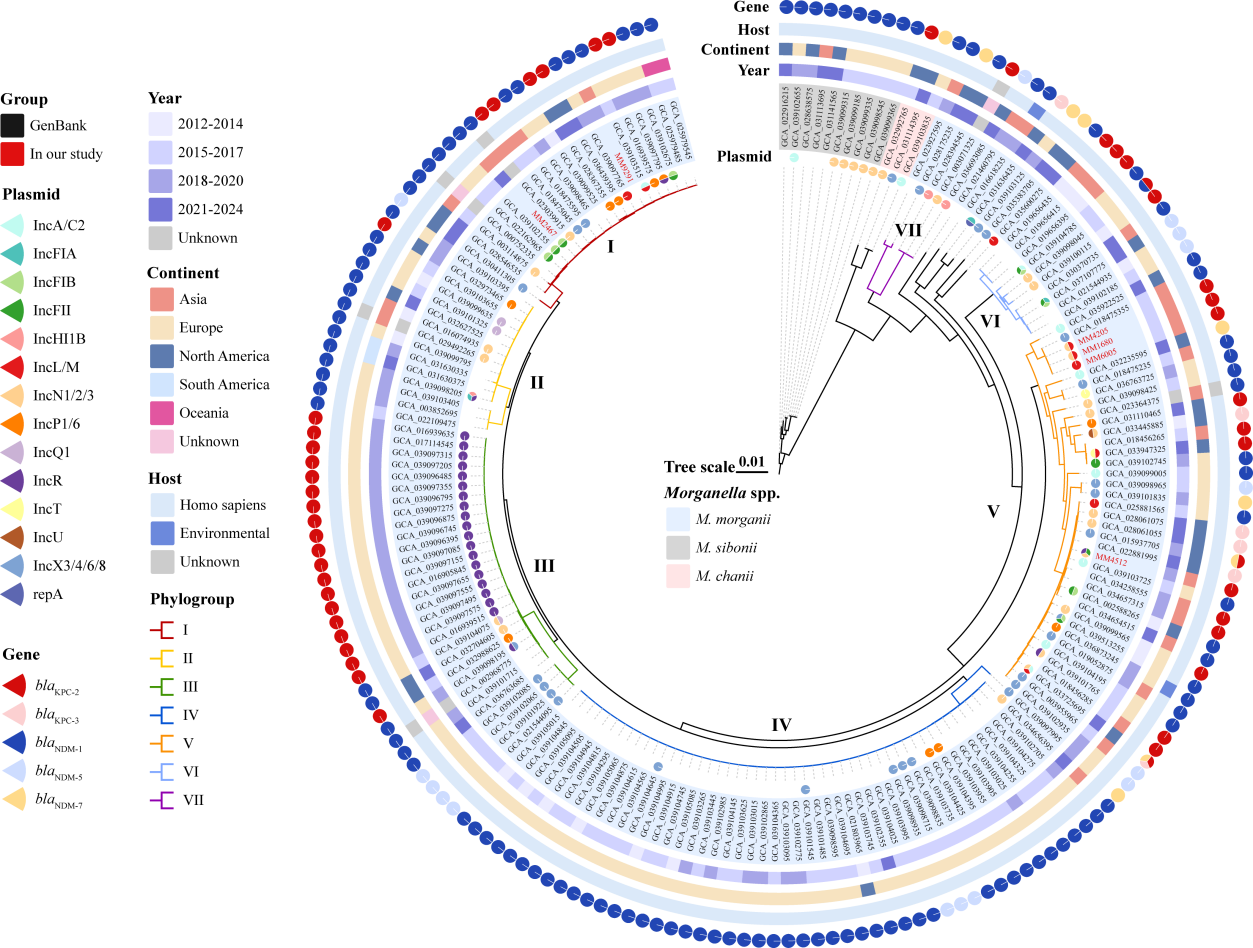
Phylogenetic tree of 190 global CRMs with *bla*_KPC_ or *bla*_NDM_. A phylogenetic tree was constructed using Roary and FastTree from Snippy and further visualized using ChiPlot. Based on the FastANI tool, species were delineated according to an average nucleotide identity (ANI) greater than 95%. Carbapenemases produced are indicated by colored pie charts. Phylogroups (I–VII) represent the seven clones of *M. morganii*.

Subsequently, we constructed a phylogenetic tree for the 190 CRMs (Fig. 7). In this phylogenetic tree, two species, *M. morganii* (*n* = 177) and *M. sibonii* (*n* = 10), diverged earliest and independently clustered into two major phylogroups, indicating that these two complexes have relatively distinct evolutionary histories. *M. chanii* (*n* = 3) is an emerging complex that has evolved within *M. morganii*. As anticipated, there was consistency between the results of the ANI species identification and phylogenetic analysis. The population of *M. morganii* (*n* = 177) harboring *bla*_KPC_ or *bla*_NDM_ was significantly larger than that of the other two species. *M. morganii* exhibited a highly clonal structure with seven phylogroups (I–VII), in which our isolates were in phylogroups I and V. The expansion of phylogroups has shown certain regional specificities. For example, phylogroups III and IV are primarily concentrated in Europe, with few occurrences in Asia. A single *bla*_KPC-2_ or *bla*_NDM-1/5_ gene was present in *M. morganii* identified in phylogroups I–IV from 2015 to 2020. In addition, over 20 plasmids were identified using PlasmidFinder in ABRicate. Furthermore, a significant correlation between IncR plasmids and *bla*_KPC-2_ was observed in the phylogroup III isolates.

## DISCUSSION

A total of 986 *Morganella* spp. isolates were classified into six distinct species. Three relatively rare species (*M. kristinii*, *M. psychrotolerans,* and *M. laugraudii*) were found exclusively in animals and food products, indicating that there are certain biological barriers to the interhost transmission of these bacterial strains (Fig. S2). Moreover, the *bla*_KPC_ and *bla*_NDM_ genes are also present only in three common species (*M. morganii*, *M. sibonii*, and *M. chanii*), indicating that the horizontal or vertical transmission of resistance genes may confer some species with "epidemic" clones (25). Notably, there was a significant difference in the number of resistance genes between the epidemic (mean = 7.86) and non-epidemic clones (mean = 0.17) of *Morganella* spp. (*P*_t_ <0.0001), respectively (Fig. S3). However, the intrinsic mechanisms that enable some *Morganella* spp. to act as reservoirs for resistance genes warrant further investigation. Certainly, the results may exhibit certain biases owing to the limited number of sequenced genomes. Therefore, widespread adoption of WGS is essential for advancing our understanding of the complexities of species evolution.

In this study, the CRMM isolates were resistant to third- and fourth-generation cephalosporins and carbapenems, which are commonly used clinically to treat infections caused by *Enterobacterales* (26). The AST results suggest that CZA is a viable option for treating KPC-producing CRMM isolates, but is ineffective against NDM-producing strains. The prevalence of NDM-1-producing *M. morganii* presents a great challenge for clinical treatment not only because of its natural resistance to colistin and tigecycline (27) but also because of its acquired resistance to CZA, which is one of the last options for the treatment of carbapenem-resistant Gram-negative bacteria (28). Fortunately, CFDC exhibits potent *in vitro* antimicrobial efficacy against CRMM, presenting itself as a novel therapeutic alternative for infections caused by CRMM isolates.

The WGS revealed the widespread presence of ARGs in these CRMM strains. Plasmid diversity is the main reason for the spread of ARGs among bacteria (29, 30). More than 20 known plasmids, including IncN1/2/3 (*n* = 27), IncX3/4/6/8 (*n* = 27), IncR (*n* = 24), IncP1/6 (*n* = 10), IncFII (*n* = 10), IncA/C2 (*n* = 10), and IncL/M (*n* = 9), were discovered in 190 CRMs (Table S5). In our isolates, *bla*_KPC-2_ was found in IncN, IncL/M, and IncFII-R, whereas *bla*_NDM-1_ was located in IncFII-FIB and an unknown plasmid, which is consistent with other studies (29–31). Most of these plasmids are transferable and pose a considerable risk of horizontal transmission between species. Except for one plasmid, which was rapidly lost without selective pressure, most plasmids carrying resistance genes could persist stably regardless of the presence or absence of antibiotic stress. This stability significantly increases the likelihood of CRMM evolving into "superbug" isolates and suggests the potential for persistent infections in patients.

In addition to plasmids, transposons are pivotal in the evolution of bacteria and dissemination of functional genetic cargo, such as genes conferring antimicrobial resistance (32). Through database screening and sequence comparison, we found that the transmission of *bla*_KPC-2_ was mediated by four transposons, ΔTn*801*, ΔTn*21*, ΔTn*5403*, and ΔTn*4401*, in *M. morganii* (29, 33–37). Notably, the horizontal transfer of Tn*6296* was crucial for the emergence and rapid transmission of *bla*_KPC_ in our strains. Most plasmids were situated on truncated ΔTn*6296* based on IS*26* (38). It is noteworthy that they all had a common core structure, Δ*tnpR*-IS*Kpn27-bla*_KPC-2_-IS*Kpn6*, which is consistent with the findings of our previous study on *Klebsiella aerogenes* (39). IS*26* is classified as a pseudocomplex transposon, in which two small DNA segments flanking IS*26* are created by cointegration. Previous studies have shown that IS*26* is involved in the spread and amplification of *bla*_KPC_ and may generate tandem repeat sequences through homologous recombination (40). Interestingly, we discovered three isolates of *M. morganii* collected in the ICU that carried plasmids, illustrating the evolutionary process of the IncL/M plasmid acquiring *bla*_KPC-2_ via a mobile sequence mediated by IS*26* over time. These data strongly suggest that IS*26* is a key factor in the emergence and rapid transmission of *bla*_KPC_. In addition, the Tn*125* transposon seems to have played an important role in the early plasmid-mediated transfer of *bla*_NDM_ in CRMM isolates but has been overtaken in recent years by other elements, including IS*26* and the IS*CR* family of insertion sequences (30). There was a common core structure, *bla*_NDM_-*bla*_MBL_-*trpF*, which not only carried other resistance genes transferred among plasmids but also inserted two or more copies into chromosomes mediated by IS*CR1/3*. This suggests that the IS*CR* element plays a significant role in the later stages of *bla*_NDM_ dissemination (30).

Through phylogeographic analysis, we further discovered that Europe has emerged as the continent with the highest emergence of CRMs carrying *bla*_KPC_ or *bla*_NDM_, which was largely attributed to a recent multicenter study (11). Out of the *Morganella* spp. obtained from NCBI, the sequencing resources are predominantly biased towards a few countries, and there is a lack of assessment regarding the epidemiological trends in some middle- to low-income countries, which needs further investigation. As shown by the SNPs and phylogenetic tree, we observed clonal transmission of CRMM, which was independent of geographic location, time, and host. Nevertheless, some CRMM strains in our hospital currently exhibit a pattern of clonal dissemination. Therefore, it is necessary to establish a relevant nosocomial infection surveillance program, including the construction of a multilocus sequence typing (MLST) system and monitoring the expansiveness and harmfulness of clinical mutant strains, to prevent the emergence of high-risk epidemic clones (41).

### Conclusions

Our 7-year surveillance and genomic analysis revealed critical insights into the genetic characteristics and transmission dynamics of CRMM isolates producing KPC or NDM. The identification of mobile genetic elements, such as Tn*6296* and Tn*125*, underscores their role in the horizontal transfer of resistance genes, facilitating the spread of *bla*_KPC-2_ and *bla*_NDM-1_. Furthermore, our global genomic epidemiology study elucidated the spatial–temporal distribution of *Morganella* spp., providing a comprehensive understanding of their evolutionary trajectories. Given the escalating threat posed by these "epidemic" clones, it is imperative to enhance surveillance and implement effective control measures in clinical settings.

## MATERIALS AND METHODS

### Clinical isolation and resistance gene screening

From January 2016 to December 2022, *M. morganii* isolates identified as CRE through clinical testing were collected from a tertiary hospital in Hangzhou, China. Strain identification was performed using MALDI-TOF MS (Sysmex-Biomeirers, France). These strains were screened for carbapenem resistance genes (*bla*_KPC_ and *bla*_NDM_) using colloidal gold immunochromatography (NG-Test CARBA 5, FOSUM DIAGNOSTICS) containing *bla*_KPC_, *bla*_NDM_, *bla*_VIM_, *bla*_IMP_, and *bla*_OXA-48_ for rapid detection (42), polymerase chain reaction (PCR), and Sanger sequencing for verification, as previously described (43, 44). All isolates were stored at −80°C in a glycerin bouillon for further investigation.

### Antimicrobial susceptibility testing

AST of nine antimicrobial agents, including meropenem (MEM), imipenem (IPM), ertapenem (ETP), ciprofloxacin (CIP), cefepime (FEP), ceftazidime (CAZ), ceftazidime-avibactam (CZA), and amikacin (AMK) was performed using a broth microdilution method with cation-adjusted Mueller–Hinton broth (CAMHB), while cefiderocol (CFDC) was tested with iron-depleted CAMHB. The testing conditions and results were interpreted by the Clinical and Laboratory Standards Institute (7). *Escherichia coli* ATCC 25922 was used as quality control.

### Conjugative transfer experiments

To explore the transferability of plasmids harboring *bla*_KPC-2_ or *bla*_NDM-1_, a conjugative transfer assay was performed using recipient *E. coli* J53 (Azi^R^). First, monoclonal colonies of donor and recipient bacteria were collected into 2 mL of Luria–Bertani (LB) broth, expanded to the logarithmic growth phase, mixed at a ratio of 1:1, and cultured on MH plates with a filter membrane (0.22 µm) overnight. Transconjugants were screened on MH agar containing sodium azide (300 µg/mL) and ampicillin (100 µg/mL). Finally, the recovered transconjugants were confirmed by PCR, MALDI-TOF MS, and MIC results.

### Stability assays and growth rate determination

The stability of plasmids containing *bla*_KPC-2_ or *bla*_NDM-1_ in the transconjugants was assessed using a passage experiment in the absence of antibiotics, as previously described (45). Growth rate determination was used to evaluate the fitness cost of the transconjugants (46). Three independent cultures per transconjugant were grown overnight in LB broth (with 300 µg/mL azide and 100 µg/mL ampicillin), diluted 1:1,000 in LB broth without antibiotics, and aliquoted into a flat-bottom 100-well plate in triplicate. The plates were incubated at 37°C with shaking at 200 rpm. OD_600_ curves were measured every 5 min for 20 h using a Bioscreen C MBR machine (Oy Growth Curves Ab Ltd., Finland). The relative growth rate based on the OD_600_ curves was calculated using R script.

### Whole-genome sequencing and genomic analysis

WGS was conducted using the Illumina HiSeq and Nanopore MinION platforms at Zhejiang Tianke (Hangzhou, China) as previously described (46). Complete genome sequences were assembled using the hybrid assembly tool Unicycler 0.4.8 (47) and annotated using RAST (https://rast.nmpdr.org/) (48). ResFinder (49) and PlasmidFinder (50) from the Center of Genomic Epidemiology (https://www.genomicepidemiology.org/services/) were used to identify antibiotic resistance genes and plasmid types, respectively. BLAST from NCBI (https://blast.ncbi.nlm.nih.gov/Blast.cgi/) was used to find similar sequences of plasmids and genes (51). Sequence comparisons were performed using BLASTn v2.4.0 (52) and visualized using Easyfig v2.2.3 (53) and Proksee (https://proksee.ca/).

### Global phylogeographic and phylogenetic analyses of *Morganella* spp.

To further monitor the global phylogeography of CRMs carrying *bla*_KPC_ or *bla*_NDM_, we assessed all available 962 genomes of *Morganella* spp. in GenBank (https://www.ncbi.nlm.nih.gov/datasets/genome, accessed on 20 June 2024). Species were delineated based on an average nucleotide identity (ANI) >95% using FastANI (https://github.com/ParBLiSS/FastANI) (Table S3) (54, 55). A total of 184 (19.13%, 184/962) CRMs harboring *bla*_KPC_ or *bla*_NDM_ were obtained through ARG analysis using ABRicate (https://github.com/tseemann/abricate) (56). Utilizing the existing Python-written biosample code (https://github.com/stajichlab/biosample_metadata), we performed a batch search for biological sample information of the isolates. The phylogenetic tree was constructed using Roary (57) and FastTree (58) and further visualized using ChiPlot (https://www.chiplot.online/) (59). Heatmaps were utilized for the visualization of single nucleotide polymorphisms (SNPs) through TBtools-II (60). Adobe Illustrator v27.9.1 was used to map the global geographic distribution.

### Nucleotide sequence accession numbers

The complete chromosome and plasmid sequences of the six isolates (including four *bla*_KPC-2_-positive isolates and two *bla*_NDM-1_-positive isolates) were submitted to GenBank, with the accession numbers listed in Table S2.

## SUPPLEMENTAL MATERIAL

Supplemental figures spectrum.02476-24-s0001.pdf Fig. S1 to S3.

Supplemental tables spectrum.02476-24-s0002.xlsx Tables S1 to S5.

ASM does not own the copyrights to Supplemental Material that may be linked to, or accessed through, an article. The authors have granted ASM a non-exclusive, world-wide license to publish the Supplemental Material files. Please contact the corresponding author directly for reuse.
