# Supplementary material for: Carbapenem-resistant Morganella morganii carrying blaKPC-2 or blaNDM-1 in the clinic: one-decade genomic epidemiology analysis
Source: Microbiol Spectr. 2025 Mar 3;13(4):e02476-24. doi: 10.1128/spectrum.02476-24 (PMC11960177; doi:10.1128/spectrum.02476-24)
Supplement: Supplemental figures — Fig. S1 to S3. [file spectrum.02476-24-s0001.pdf]

Supplemental Figures

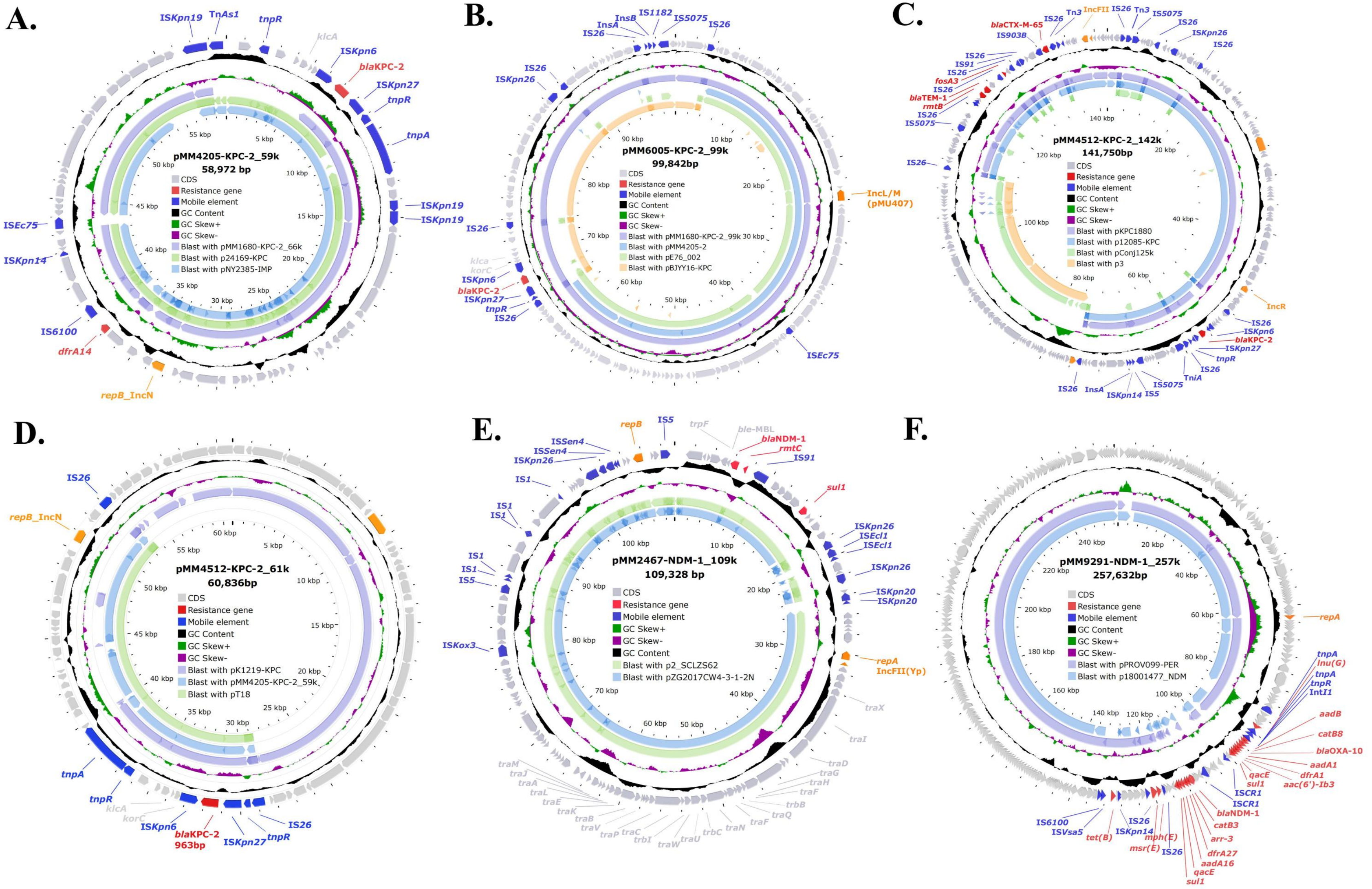

Fig. S1. Circular sequence alignment of plasmids bearing *bla*$_{KPC-2}$ or *bla*$_{NDM-1}$. The plasmids originated from two sources: our study and other similar plasmids available from the NCBI database through BLAST. **(A)** Both pMM4205-KPC-2_59k (58,972 bp) and pMM1680-KPC-2_66k (66,924 bp), were highly similar to p24169-KPC (MN891676, 69k) (100% identity and 96% coverage) from clinical *Klebsiella pneumoniae*, as well as to pNY2385-IMP (CP096922, 62k) (100% identity and 93% coverage) from *Citrobacter freundii*. **(B)** The pMM6005-KPC-2_99k (99,842 bp), a large recombinant plasmid, showed significant similarity to pMM1680-KPC-2_99k based on the IncL/M plasmid with IS*26*-mediated mobile sequences. The MM4512 strain hosted double *bla*$_{KPC-2}$ in two separate plasmids, one of which was pMM4512-KPC-2_142k (141,750 bp) **(C)**, classified as an IncFII-R recombinant plasmid. This complex plasmid contains a variety of mobile elements and several resistance genes, including *bla*$_{KPC-2}$, *bla*$_{CTX-M-65}$, *bla*$_{TEM-1B}$, *fosA3* and *rmtB*. It was similar to pKPC1880 (CP061347, 169k) (100% identity and 74% coverage) and p12058-KPC (MN842292, 142k) (100% identity and 73% coverage) from *K. pneumoniae*. A type IV secretion system (T4SS), similar to p3 (CP132686, 35k) from *K. pneumoniae* and pConj125k (MK033499, 125k) from *E. coli*, was inserted by IS*26*. Additionally, the pMM4512-KPC-2_61k (60836 bp) **(D),** carrying *bla*$_{KPC-2}$ variant (963 bp) with *bla*$_{KPC-2}$_1 (AY034847, VCV70871) (93% identity and 99% coverage), showed the highest similarity to pK1219-KPC (CP137007, 140k) (100% identity and 76% coverage) from *Citrobacter koseri* and pMM4205-KPC-2_59k (IncN) in our study. **(E)** The pMM2467-NDM-1_109k (109,328 bp) was highly similar to p2_SCLZS62 (CP082170, 111k) (100% identity and 97% coverage)

from *Raoultella planticola* and pZG2017CW4-3-1-2N (CP065352, 108k) (100% identity and 95% coverage) from *K. pneumoniae*. **(F)** The pMM9291-NDM-1_257k (257,632 bp), a recombined plasmid, was similar to pPROV099-PER (CP120536, 259k) (100% identity and 91% coverage) and p18001477_NDM (CP098041, 273k) (100% identity and 91% coverage) from *Providencia rettgeri*.

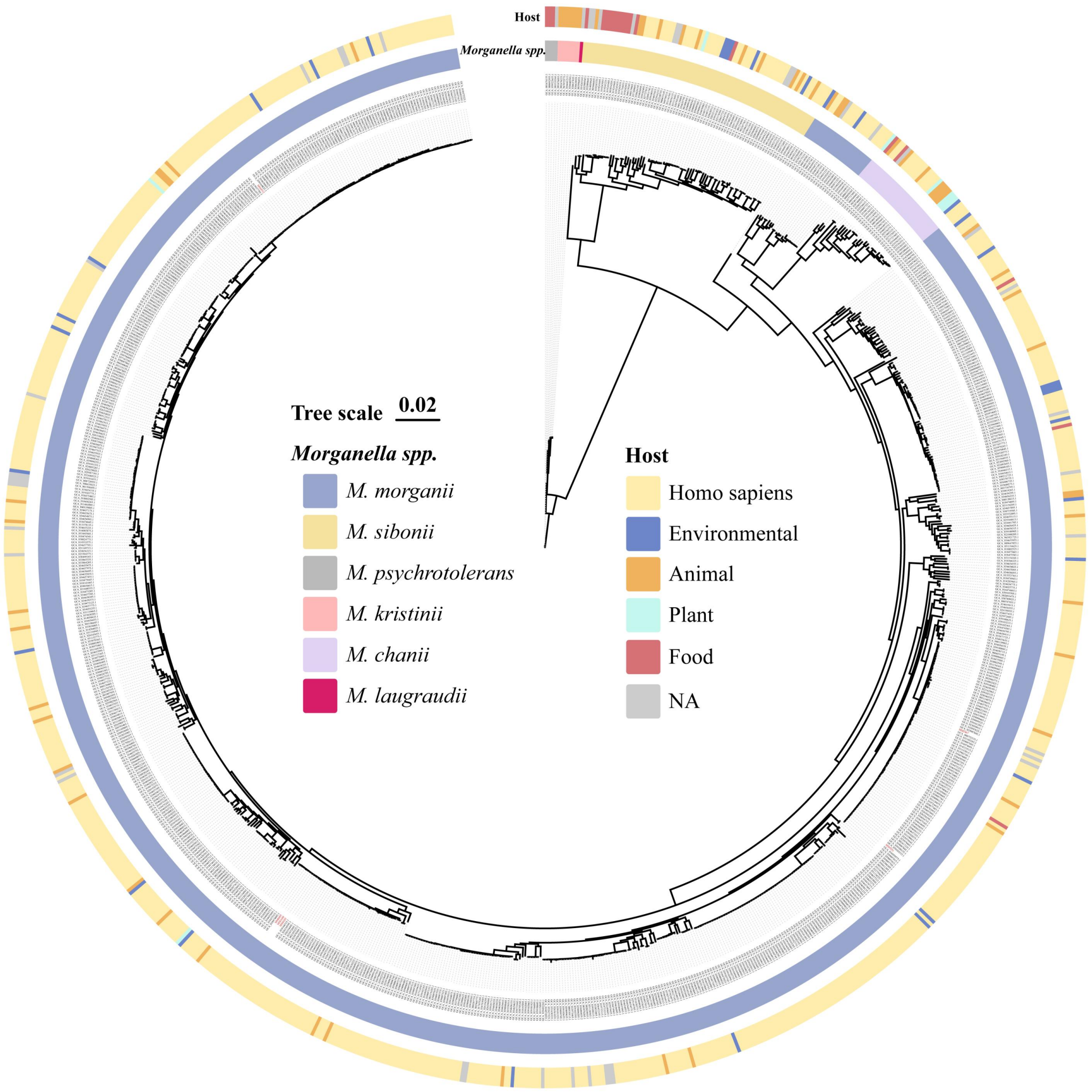

Fig. S2. Phylogenetic tree of global 968 *Morganell* spp. isolates. Employing the Fastani identification method, a genomic relatedness threshold of ANI (Average Nucleotide Identity) greater than 95% was established to delineate homologous species. This criterion successfully classified all 968 ***Morganella* spp.** into the 6 species. **Reference Sequences: *Morganella morganii*** (GCA_006094455.1), ***Morganella sibonii*** (GCA_039099315.1), ***Morganella chanii*** (GCA_000633515.1), ***Morganella kristinii*** (GCA_001676055.1), ***Morganella laugraudii*** (GCA_003996855.1), ***Morganella psychrotolerans*** (GCA_001676155.1).

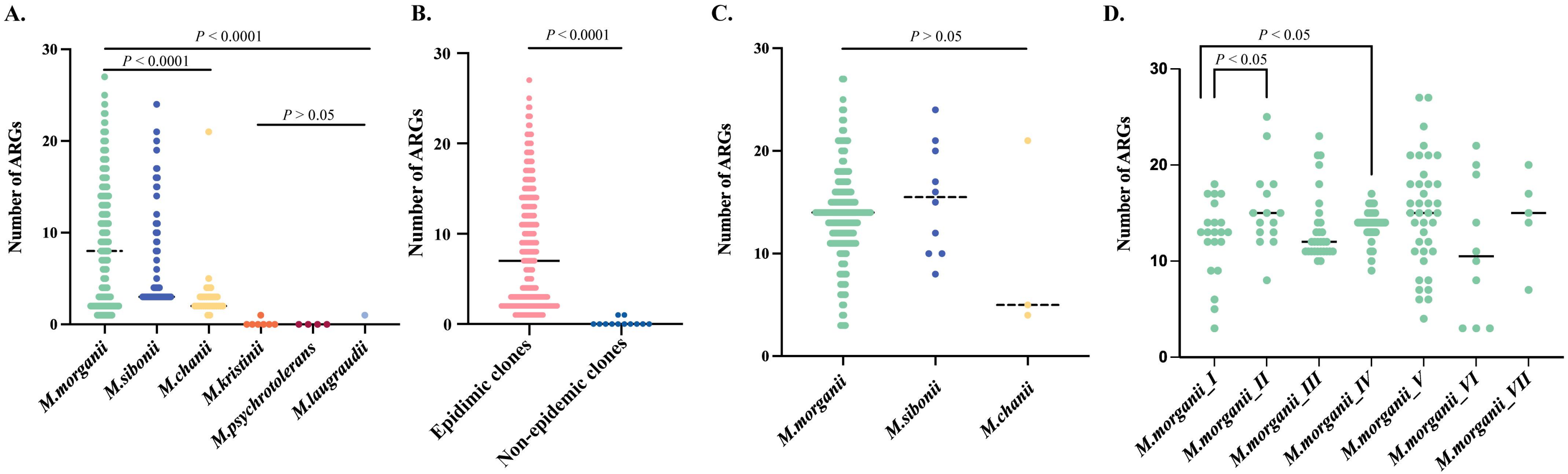

Fig. S3. Comparative analysis of ARGs quantities among different *Morganella* spp. (A) Among 968 *Morganella* spp. isolates, the number of ARGs in epidimic clones (*M. morganii*, *M. sibonii* and *M. chanii*) was greater than in non-epidemic clones (*M. kristinii*, *M.psychrotolerans* and *M. laugraudii*), as determined by ANOVA and two sample *t*-test **(B)**. **(C)** Three types of CRMs producing KPC or NDM did not exhibit significant differences in the number of ARGs. **(D)** Among 177 *M. morganii* bearing *bla*$_{\text{KPC}}$ or *bla*$_{\text{NDM}}$ genes, there were differences in the number of resistance genes among some phylogroups.
